# Human trafficking and health: a cross-sectional survey of NHS professionals’ contact with victims of human trafficking

**DOI:** 10.1136/bmjopen-2015-008682

**Published:** 2015-07-31

**Authors:** Claire Ross, Stoyanka Dimitrova, Louise M Howard, Michael Dewey, Cathy Zimmerman, Siân Oram

**Affiliations:** 1 King's College London, David Goldberg Centre, London, UK; 2 London School of Hygiene & Tropical Medicine, London, UK

**Keywords:** Human trafficking, Identification, Referral, Training

## Abstract

**Objectives:**

(1) To estimate the proportion of National Health Service (NHS) professionals who have come into contact with trafficked people and (2) to measure NHS professionals’ knowledge and confidence to respond to human trafficking.

**Design:**

A cross-sectional survey.

**Setting:**

Face-to-face mandatory child protection and/or vulnerable adults training sessions at 10 secondary healthcare provider organisations in England, and meetings of the UK College of Emergency Medicine.

**Participants:**

782/892 (84.4%) NHS professionals participated, including from emergency medicine, maternity, mental health, paediatrics and other clinical disciplines.

**Measures:**

Self-completed questionnaire developed by an expert panel. Questionnaire asks about prior training and contact with potential victims of trafficking, perceived and actual human trafficking knowledge, confidence in responding to human trafficking, and interest in future human trafficking training.

**Results:**

13% participants reported previous contact with a patient they knew or suspected of having been trafficked; among maternity services professionals this was 20.4%. However, 86.8% (n=679) reported lacking knowledge of what questions to ask to identify potential victims and 78.3% (n=613) reported that they had insufficient training to assist trafficked people. 71% (n=556), 67.5% (n=528) and 53.4% (n=418) lacked confidence in making appropriate referrals for men, women and children, respectively, who had been trafficked. 95.3% (n=746) of respondents were unaware of the scale of human trafficking in the UK, and 76.5% (n=598) were unaware that calling the police could put patients in more danger. Psychometric analysis showed that subscales measuring perceived knowledge, actual knowledge and confidence to respond to human trafficking demonstrated good internal consistency (Cronbach's αs 0.93, 0.63 and 0.64, respectively) and internal correlations.

**Conclusions:**

NHS professionals working in secondary care are in contact with potential victims of human trafficking, but lack knowledge and confidence in how to respond appropriately. Training is needed, particularly for maternity staff, on how to identify and respond to victims’ needs, including through making safe referrals.

**Strengths and limitations of this study**

- A survey was conducted in multiple sites across England (10 secondary healthcare provider organisations plus meetings of the UK College of Emergency Medicine).
- Sample includes health professionals working across a range of disciplines and grades.
- Psychometric analyses indicate the study instrument has good internal consistency, good correlation among theoretical constructs, and good discriminative characteristics in relation to previous human trafficking training.
- A survey was conducted in secondary care only; findings may not be generalisable to primary care.
- Study sites were located in police force areas with higher reported victims of trafficking (5 or more in the year to 31 December 2012): staff, and the institutions surveyed, may not be typical of the National Health Service.

## Background

Human trafficking is the recruitment and movement—most often by means of coercion, deception or abuse of vulnerability—for the purposes of exploitation.^1^ Victims of trafficking are moved across and within international borders to be exploited through forced sex work, domestic servitude, and numerous labour settings including agriculture, car washing, construction and factory work. Human trafficking is believed to affect every country in the world, either as countries of origin, transit or destination, with recent estimates putting the scale of the crime at 2.5 million people worldwide.^2^

Victims of human trafficking experience high levels of abuse including physical violence (being hit, kicked and assaulted with weapons), sexual violence (rape and forced participation in sexual acts), psychological violence (including threats to self and to family, surveillance, humiliation, intimidation), economic restrictions (including confiscation of earnings, restriction of access to funds), and other controlling behaviours, including confiscation of passport and other identity documents, and threats to report the victim to immigration, police and child welfare authorities.^3–5^ High levels of physical and mental health problems have been reported among victims of human trafficking, including non-specific symptoms such as headache, back pain, stomach pain and dizziness, and mental disorders such as depression, anxiety and post-traumatic stress disorder.^6–8^ However, little is known about the extent to which healthcare professionals come into contact with victims of trafficking, or about their knowledge and readiness to identify victims, to make appropriate referrals, and to provide clinical care.

In the USA, a cross-sectional survey of 180 emergency department staff found that although 79% knew what trafficking was, only 27% thought it was a problem that affected their patient population, and just 6% believed that they had ever treated a victim of trafficking.^9^ Also, 95% had received no formal training on responding to human trafficking, and the majority reported being hesitant or not confident in their ability to identify or correctly treat a trafficking victim who presented to the emergency department. A pretraining survey of 178 healthcare professionals attending human trafficking training in the Middle East, Caribbean and Central America reported generally high levels of knowledge regarding the health outcomes associated with human trafficking, but found that many healthcare professionals lacked awareness of their role in responding to victims of human trafficking.^10^ In the UK, a qualitative study of seven healthcare professionals working with female victims of trafficking for sexual exploitation found that staff reported difficulties with building trusting relationships with trafficked patients and with coping with the emotional burden of women's needs and experiences.^11^ To the best of our knowledge, no previous research has investigated whether UK health professionals come into contact with victims of human trafficking, their knowledge and readiness to respond to human trafficking, or their training needs.

The objectives of this study were^1^ to estimate the proportion of National Health Service (NHS) professionals in England who have come into contact with victims of trafficking;^2^ to measure NHS professionals’ knowledge and confidence in responding to human trafficking (including identification, referral and clinical care).

## Method

### Design

Cross-sectional survey, conducted in England between August 2013 and April 2014.

### Sample

Healthcare professionals attending face-to-face mandatory child protection and/or vulnerable adults training sessions at 10 secondary healthcare provider organisations located in areas in which five or more victims of trafficking had been identified by police prior to 31 December 2012 (Birmingham Women's Hospital NHS Foundation Trust, Cambridge University Hospital NHS Foundation Trust, Croydon Health Services NHS Trust, East Kent Hospitals University NHS Foundation Trust, Greater Manchester West Mental Health NHS Foundation Trust, Guy's and St Thomas’ NHS Foundation Trust, Hillingdon Hospitals NHS Foundation Trust, Homerton University Hospital NHS Foundation Trust, King's College Hospital NHS Foundation Trust, and South London and Maudsley NHS Foundation Trust) and at meetings of the College of Emergency Medicine. No incentives for participation were offered.

### Instrument

The PROTECT survey assesses healthcare professionals’ levels of knowledge and attitudes towards human trafficking (see online supplementary information). Existing human trafficking and violence clinician surveys were reviewed and survey items developed and adapted by LMH, CZ and SO. Proposed survey items were reviewed by the PROTECT Project Steering Group, a group of academics and clinical academics with expertise in human trafficking, health education, emergency medicine, psychiatry, midwifery, sexual health and social work (see Acknowledgements section), and piloted with healthcare professionals (n=7), and with King's College London health visitor students (n=40). Following revisions, the questionnaire comprised 51 items over six sections: (1) background information (6 items); (2) experience of training and responding to human trafficking (9 items); (3) perceived knowledge of human trafficking (9 items); (4) actual knowledge about human trafficking (19 items); (5) responding to human trafficking (13 items) and (6) interest in attending human trafficking training (2 items). The PROTECT questionnaire is self-administered, takes approximately 10 min to complete, and is designed to be relevant across a range of clinical disciplines and settings.

### Procedure

Staff attending mandatory, face-to-face training sessions on child protection and/or safeguarding vulnerable adults were asked to participate in the study. Study researchers attended the training sessions and described the study aims and procedures and answered questions about the study before participating professionals completed the self-administered questionnaire.

### Analysis

Data were entered into an MS Access Database and analysed using STATA V.12. Descriptive statistics (frequencies, percentages, means, medians, SDs and IQRs) were calculated. An exploratory factor analysis was conducted to evaluate the psychometric properties of the PROTECT questionnaire. Background information, questions about interest in future human trafficking training, and open-ended questions were not included in the psychometric analysis, and negatively worded items were reverse coded. Maximum Likelihood Analysis with oblique rotation used for factor analysis, after the Kaiser-Meyer Olkin (KMO) measure indicated that the sample was adequate for factor analysis (KMO=0.85). Cronbach's α was used to assess internal construct reliability within the identified scales. Kuder-Richardson was used to assess item difficulty in the knowledge subscale. Correlation analysis and multiple regressions were used to assess internal consistency of the PROTECT questionnaire and the discriminative characteristics of the scales with respect to participants who had and had not previously received training on human trafficking.

### Ethics

Local approval was provided by each of the participating Trusts. Participants were provided with verbal and written information about the study, and provided informed consent to participate. Each participant was provided with a unique identification number which was used when managing the research data.

### Role of the funding source

The funder had no role in the design or conduct of the study, collection, management, analysis and interpretation of the data or writing of the report.

## Results

### Sample characteristics

A total of 892 healthcare professionals were invited to participate in the study, of whom 782 (84.4% response rate) consented. Four-fifths (80.6%) of participants were women, and nearly three-fifths (57%) had qualified in the past 10 years. Table 1 provides further details of the sociodemographic and professional characteristics of the sample.

**Table 1:** Sociodemographic and employment characteristics of the sample (n=782)*

	n=782 (%)
Gender	
Male	152 (19.4)
Female	630 (80.6)
Age (years)	
19–24	95 (12.1)
25–30	176 (22.5)
31–40	158 (20.2)
41–50	108 (13.8)
51–60	51 (6.5)
61–70	12 (1.5)
Ethnicity	
White	553 (70.7)
Mixed	26 (3.3)
Asian/British Asian	72 (9.2)
Black/African/Caribbean	72 (9.2)
Other	9 (1.1)
Clinical discipline	
Clinical therapies†	62 (7.9)
Emergency medicine	89 (11.3)
Maternity	137 (17.5)
Mental health	174 (22.3)
Paediatrics	52 (6.6)
Other clinical discipline‡	182 (23.3)
Non-clinical§	24 (3.1)
Current NHS role	
Doctor/dentist	124 (15.8)
Healthcare assistant/porter	35 (4.5)
Midwife	65 (8.3)
Nurse	265 (33.4)
Psychologist/counsellor	34 (4.3)
Student	48 (6.1)
Technical support/administration	33 (4.2)
Other¶	126 (16.1)
Year of qualification	
1965–1974	5 (0.6)
1975–1984	37 (4.7)
1985–1994	81 (10.3)
1995–2004	114 (14.5)
2005–2014	331 (42.3)
Not yet qualified	9 (1.2)

*Owing to missing data, columns may not total to 782.

†Clinical therapies include physiotherapy, audiology, speech and language.

‡Other clinical disciplines, for example, endocrinology, haematology, infectious diseases, neurology, oncology, ophthalmology, orthopaedics, etc.

§Non-clinical includes, for example, administrators, chaplains, porters, receptionists, etc.

¶Other current NHS roles include physiotherapist, occupational therapist, speech and language therapist, audiologist dietician, social worker, operating department practitioner, radiographer, phlebotomy, pharmacist, safeguarding lead, prison link worker, chaplain.

NHS, National Health Service.

### Psychometric properties of the PROTECT questionnaire

Psychometric analyses found that the internal consistency of the questionnaire subscales was good or higher: Cronbach's αs for perceived knowledge of human trafficking, actual knowledge of human trafficking, and responding to human trafficking were 0.93 (95% CI 0.92 to 0.94), 0.63 (95% CI 0.59 to 0.66) and 0.64 (95% CI 0.60 to 0.68), respectively. Maximum likelihood factor (MLF) analysis of the perceived knowledge of human trafficking subscale revealed a dominant one-factor solution, with all items loading onto the one factor weighted higher than 0.71, and with uniqueness below 0.30. MLF analysis of the responding to human trafficking subscale similarly showed a one-factor solution. Kuder-Richardson analysis of the knowledge section suggested that 16 of the 19 questions were difficult (rated >0.51), with an average rating of KR=0.71. MLF analysis demonstrated a two-factor solution after dropping two items (1: diabetes is not likely to be associated with situations of human trafficking and 2: coronary heart disease is not likely to be associated with situations of human trafficking), one loading onto symptom-based questions and one loading onto questions about general knowledge of human trafficking. Further analyses found that perceived knowledge was significantly correlated with knowledge (R=0.1305, p=0.001) and with responding to trafficking (R=0.3497, p=0.001), and that experience of human trafficking training was correlated with perceived knowledge (R=0.385, p<0.001) and responding to human trafficking (R=0.136, p<0.001).

### Contact with trafficked people

Thirteen per cent of respondents reported that they had previously been in contact with a patient whom they knew or suspected had been trafficked (n=102, 13.0%), ranging from 6.7% (5/75) to 20.8% (15/72) across study sites. Among professionals working in maternity services, the proportion reporting prior contact with a potential victim of trafficking rose to 20.4% (28/137) (table 2). Among those professionals reporting previous contact with trafficked people, 25% (25/102) reported that their knowledge or suspicions arose because of disclosure by another professional involved in their care, 32% (33/102) because of disclosure by the patient, and 11% (11/102) for other reasons including police involvement and the child being in foster care. However, nearly a third of participants reporting previous contact with a patient they knew or suspected of having been trafficked did not report why their suspicions had arisen (33/102). Survey participants were asked what were the most important signs or indications that would suggest to them that a patient may have been trafficked: most commonly cited were indicators of psychological distress (23.6%), indicators of physical abuse (12.4%), language barrier, and late presentation or poor engagement with healthcare (both 8.5%).

**Table 2:** Prior contact with potential victims of trafficking (n=782)*

	No prior contact, n (%) N=680	Prior contact, n (%) N=102
Clinical discipline		
Clinical therapies†	60 (96.8)	2 (3.2)
Emergency medicine	75 (84.3)	14 (15.7)
Maternity	109 (79.6)	28 (20.4)
Mental health	149 (86.1)	24 (13.9)
Paediatrics	43 (82.7)	9 (17.3)
Other clinical‡	164 (90.6)	17 (9.4)
Non-clinical§	21 (87.5)	3 (12.5)
Current NHS role		
Doctor/dentist	100 (83.3)	20 (16.7)
HCA/porter	34 (97.1)	1 (2.9)
Midwife	40 (61.5)	25 (38.5)
Nurse	244 (92.1)	21 (7.9)
Psychologist/counsellor	28 (82.4)	6 (17.6)
Student	41 (85.4)	7 (14.6)
Technical support/administration	29 (87.9)	4 (12.1)
Other¶	111 (88.1)	15 (11.9)
Reason for suspicion		
Patient disclosure	–	33 (32.4)
Professional disclosure	–	25 (24.5)
Other**	–	11 (10.8)
Not specified	–	33 (32.4)

*Owing to missing data, columns may not total to 782.

†Clinical therapies include physiotherapy, audiology, speech and language.

‡Other clinical disciplines, for example, endocrinology, haematology, infectious diseases, neurology, oncology, ophthalmology, orthopaedics, etc.

§Non-clinical includes, for example, administrators, chaplains, porters, receptionists, etc.

¶Other current NHS roles include physiotherapist, occupational therapist, speech and language therapist, audiologist dietician, social worker, operating department practitioner, radiographer, phlebotomy, pharmacist, safeguarding lead, prison link worker, chaplain.

**Child was in foster care, child was not parent's, inconsistent information, unusual situation, poor communication, reduced self-esteem, police involvement, noted in records, poor living conditions.

### Perceived knowledge about human trafficking and preparedness to respond

**Figure 1:**
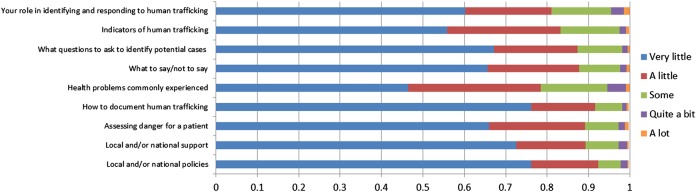
Perceived knowledge of human trafficking (n=782).

As shown in figure 1, levels of perceived knowledge about how to identify and respond to potential victims of human trafficking were low; 60.2% (n=472) of respondents reported very little knowledge regarding their role in identifying and responding to human trafficking, questions to ask to identify potential cases, what to say or not to say to a patient who has experienced human trafficking, documenting human trafficking in a medical record, assessing danger for a patient who may have been trafficked, local and national support services for trafficked people, and policies on responding to human trafficking. Perceived knowledge was slightly higher with respect to indicators of human trafficking and health problems commonly experienced by trafficked people, with 44% (n=345) and 54% (n=419), respectively, reporting at least a little knowledge of these aspects.

**Figure 2:**
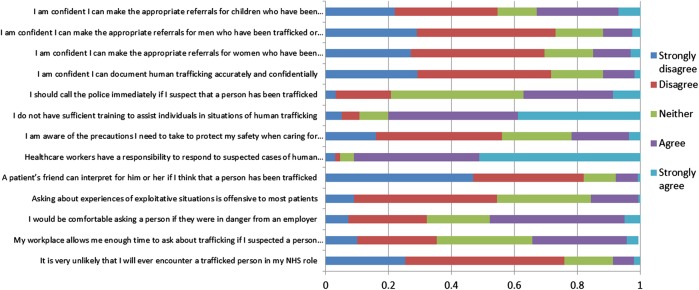
Opinions about identifying and responding to trafficked people (n=782) (NHS, National Health Service).

As shown in figure 2, although 91% (n=697) respondents agreed that healthcare professionals have a responsibility to respond to suspected cases of human trafficking, 80% (n=613) reported that they had not received sufficient training to enable them to assist individuals in such situations. The majority did not feel confident of making appropriate referrals for trafficked women (n=528, 69%), men (n=556, 73%), and children (n=418, 55%).

### Actual knowledge about human trafficking

Respondents were asked to answer 19 items measuring knowledge about human trafficking; the median number of correctly answered questions was 14 (IQR 12–15). Participants scored highly on questions relating to the health problems associated with human trafficking, with a median of eight of a maximum of nine points (IQR 7–9). However, 95.3% (n=746) of respondents were unaware of the scale of human trafficking, and 76.4% (n=598) were unaware that calling the police could put the patient in more danger (table 3).

**Table 3:** Actual knowledge about human trafficking (n=782)

Item	N (%) answering correctly	N (%) answering incorrectly	N (%) answering ‘don't know’
Definition of human trafficking is restricted to women and girls who have been trafficked into prostitution (FALSE)	687 (88.0)	47 (6.0)	47 (6.0)
More than 100 000 trafficked people were identified in the UK in 2010–2011 (FALSE)	34 (4.4)	266 (34.1)	480 (61.5)
The majority of women who are trafficked for prostitution were sex workers before being trafficked (FALSE)	545 (70.0)	72 (9.2)	165 (21.1)
Children who are working for relatives in domestic situations cannot really be considered ‘trafficked’ (FALSE)	631 (81.2)	31 (4.0)	115 (14.8)
Trafficking is associated with post-traumatic symptoms (TRUE)	496 (63.5)	88 (11.3)	197 (25.2)
Trafficking is associated with chronic headaches (TRUE)	196 (25.2)	169 (21.7)	413 (53.1)
There are usually evident signs that a person is in a trafficking situation (FALSE)	395 (50.8)	128 (16.5)	254 (32.7)
People who are being exploited often have difficulty reporting these situations to outsiders, especially professionals (TRUE)	720 (92.4)	7 (0.9)	52 (6.7)
Health practitioners should *not* ask trafficked people about violence that they might have suffered, as it is too traumatic for them (FALSE)	534 (68.6)	37 (4.8)	208 (26.7)
Calling the police if I suspect a patient has been trafficked could put the patient in more danger (TRUE)	178 (22.9)	271 (34.9)	327 (42.1)
Depression is not likely to be related to situations of human trafficking (FALSE)	760 (96.6)	27 (3.4)	–
Chemical burns and pesticide poisoning are not likely to be related to situations of human trafficking (FALSE)	679 (86.3)	108 (13.7)	–
Memory problems are not likely to be related to situations of human trafficking (FALSE)	697 (88.6)	90 (11.4)	–
Coronary heart disease is not likely to be related to situations of human trafficking (TRUE)	502 (63.8)	285 (36.2)	–
Diabetes is not likely to be related to situations of human trafficking (TRUE)	530 (67.3)	257 (32.7)	–
Hypothermia and dehydration are not likely to be related to situations of human trafficking (FALSE)	735 (93.4)	52 (6.6)	–
Sexually transmitted infections are not likely to be related to situations of human trafficking (FALSE)	771 (98.0)	16 (2.0)	–
Headaches are not likely to be related to situations of human trafficking (FALSE)	692 (87.9)	95 (12.1)	–
Post-traumatic stress disorder is not likely to be related to situations of human trafficking (FALSE)	769 (97.7)	18 (2.3)	–

### Training

Eight per cent (7.8%, n=63) of respondents reported that they had previously attended training on human trafficking, with a mean duration of 2.8 h (SD 5.4). Participants who had previously attended training were significantly more likely to report having had contact with a trafficked person (22/63, 34.9%) than those who had not (79/719, 11.0%) (p<0.001). Three-quarters of all respondents (n=569, 74.6%) reported that they would be interested in receiving such training in the future. The highest levels of interest were reported by professionals working in mental health (88.2%) and emergency medicine (81.6%).

## Discussion

International law requires that the UK provides victims of human trafficking with necessary medical treatment, including psychological assistance, counselling and information. This study provides the first evidence that a substantial proportion of NHS professionals come into contact with patients they know or suspect had been trafficked. Reported contact with potential victims of trafficking was highest among professionals working in maternity services, mental health, paediatrics and emergency medicine, and was higher than 10% in 8 of the 11 sites surveyed.

Most participants believed it was likely that they would come into contact with a victim of trafficking within the context of their NHS role. Yet, the overwhelming majority of participants reported that they had not received sufficient training to assist individuals in situations of human trafficking. Although actual knowledge regarding the definition of human trafficking and common health problems was generally high, our results suggest that NHS professionals are likely to experience difficulties in identifying and responding to human trafficking appropriately. In particular, they lack knowledge about how to ask about experiences of human trafficking, how and when to contact law enforcement agencies, and how to make referrals to local and national support services. Healthcare professionals should not contact law enforcement agencies or refer to support organisations without first discussing with their patient what options are available to them and without their patient's consent.^12^ Healthcare provider organisations should make available to their staff information about national and local referral options for survivors of human trafficking.

Training programmes have the potential to improve healthcare provider preparedness to identify and provide appropriate care and referrals for victims of trafficking. A number of resources have been developed, including an e-learning module for NHS professionals,^13^ and an international handbook and accompanying materials providing practical, non-clinical guidance for healthcare professionals,^12^ but to date have not been evaluated.

### Strengths and limitations

Psychometric analyses suggest that the PROTECT questionnaire has good internal consistency, good correlation among theoretical constructs, and good discriminative characteristics in relation to previous human trafficking training. The survey was conducted in multiple sites and was completed by staff working across a range of disciplines and grades, and a very high response rate (87.4%) was achieved. However, the survey was conducted in secondary care only, and may not be generalisable to primary care or to non-NHS settings. Professionals working in dentistry, sexual health and termination of pregnancy services were also under-represented. The staff and the institutions surveyed may not be typical of the NHS, as study sites were located in police force areas with higher reported victims of trafficking. As this was a cross-sectional survey, it is not possible to conclude whether our finding that participants who had previously attended training on human trafficking were more likely to report having been in contact with potential victims of human trafficking is thanks to the training having increased awareness of human trafficking or owing to participants seeking out training as a consequence of having been in contact with potential victims.

### Implications

The findings suggest that healthcare professionals would welcome information and training on human trafficking. Training programmes should focus on how to identify and respond to human trafficking, including what questions to ask, how to assess risk, and how to make appropriate referrals to support providers and law enforcement. Training is likely to be particularly relevant to professionals working in maternity services, mental health, paediatrics and emergency medicine, and to professionals working in areas in which higher numbers of victims of human trafficking have been previously identified, and in which shelter and other support services are provided to trafficked peopled. Evidence is now needed on the knowledge and experiences of NHS professionals working in primary care, and on the effectiveness of training programmes in improving the identification and referral of potential victims of human trafficking.

## Conclusions

Healthcare providers can play a critical role in efforts to tackle human trafficking, including by identifying and referring potential victims of human trafficking and by providing clinical care. NHS professionals working in secondary care are in contact with potential victims of human trafficking, but lack confidence in how to respond appropriately. Targeted training for professionals working in key clinical disciplines—maternity services, mental health, paediatrics and emergency medicine—may improve preparedness to identify and respond to potential victims of human trafficking and improve the well-being and safety of this vulnerable group.
